# The evolution of cephalic fins in manta rays and their relatives: functional evidence for initiation of domain splitting and modulation of the Wnt signaling pathway in the pectoral fin AER of the little skate

**DOI:** 10.1186/s13227-024-00233-3

**Published:** 2024-12-27

**Authors:** Emily P. McFarland, Karen D. Crow

**Affiliations:** 1 University of California, San Diego, USA; 2 San Francisco State University, San Francisco, USA

**Keywords:** Myliobatidae, Evolution, Development, Cephalic fin, AER, *Wnt3*

## Abstract

**Background:**

Batoids possess a unique body plan associated with a benthic lifestyle that includes dorsoventral compression and anteriorly expanded pectoral fins that fuse to the rostrum. The family Myliobatidae, including manta rays and their relatives, exhibit further modifications associated with invasion of the pelagic environment, and the evolution of underwater flight. Notably, the pectoral fins are split into two domains with independent functions that are optimized for feeding and oscillatory locomotion. Paired fin outgrowth is maintained during development by *Wnt3*, while domain splitting is accomplished by expression of the Wnt antagonist *Dkk1*, which is differentially expressed in the developing anterior pectoral fins of myliobatids, where cephalic fins separate from pectoral fins. We examine the evolution of this unique feature in the cownose ray (*Rhinoptera bonasus*), a member of the genus that is sister to *Mobula*.

**Results:**

Here, we provide functional evidence that DKK1 is sufficient to initiate pectoral fin domain splitting. Agarose beads soaked in DKK1 protein were implanted in the pectoral fins of little skate (*Leucoraja erinacea*) embryos resulting in AER interruption. This disruption arrests fin ray outgrowth, resembling the myliobatid phenotype. In addition, fins that received DKK1 beads exhibit interruption of *Axin2* expression, a downstream target of β-catenin-dependent Wnt signaling and a known AER marker. We demonstrate that *Msx1* and *Lhx2* are also associated with fin expansion at the AER. These results provide functional evidence for the underlying genetic pathway associated with the evolution of a novel paired fin/limb modification in manta rays and their relatives. We introduce the gas/brake pedal model for paired fin remodeling at the AER, which may have been co-opted from domain splitting in pelvic fins of cartilaginous fishes 370 million years earlier.

**Conclusions:**

The pectoral fins of manta rays and their relatives represent a dramatic remodel of the ancestral batoid body plan. The premiere feature of this remodel is the cephalic fins, which evolved via domain splitting of the anterior pectoral fins through inhibition of fin ray outgrowth. Here, we functionally validate the role of *Dkk1* in the evolution of this phenotype. We find that introduction of ectopic DKK1 is sufficient to recapitulate the myliobatid pectoral fin phenotype in an outgroup lacking cephalic fins via AER interruption and fin ray truncation. Additional gene expression data obtained via in situ hybridization suggests that cephalic fin development may have evolved as a co-option of the pathway specifying claspers as modifications to the pelvic fins, the only other known example of domain splitting in vertebrate appendages.

**Supplementary Information:**

The online version contains supplementary material available at 10.1186/s13227-024-00233-3.

## Introduction

The skates and rays (Batoidea) are ancestral jawed vertebrates that have evolved several striking body plan modifications, making them an interesting model for the evolution and development of derived morphologies. They exhibit dorsoventral compression with pectoral fins that extend anteriorly and fuse at the rostrum, creating a disk-like appearance that is, for most taxa, associated with undulatory swimming [1, 2] and a benthic lifestyle. In addition to locomotion, skates and rays use their expanded pectoral fins for prey capture behaviors, such as trapping prey against a substrate [1, 3]. Manta rays and their relatives (Myliobatidae, *sensu* [4]) are unique in that their pectoral fins are split into two functional domains (the anterior cephalic fins and the remaining pectoral fins), which are optimized for feeding and locomotion, respectively [5].

**Fig. 1:**
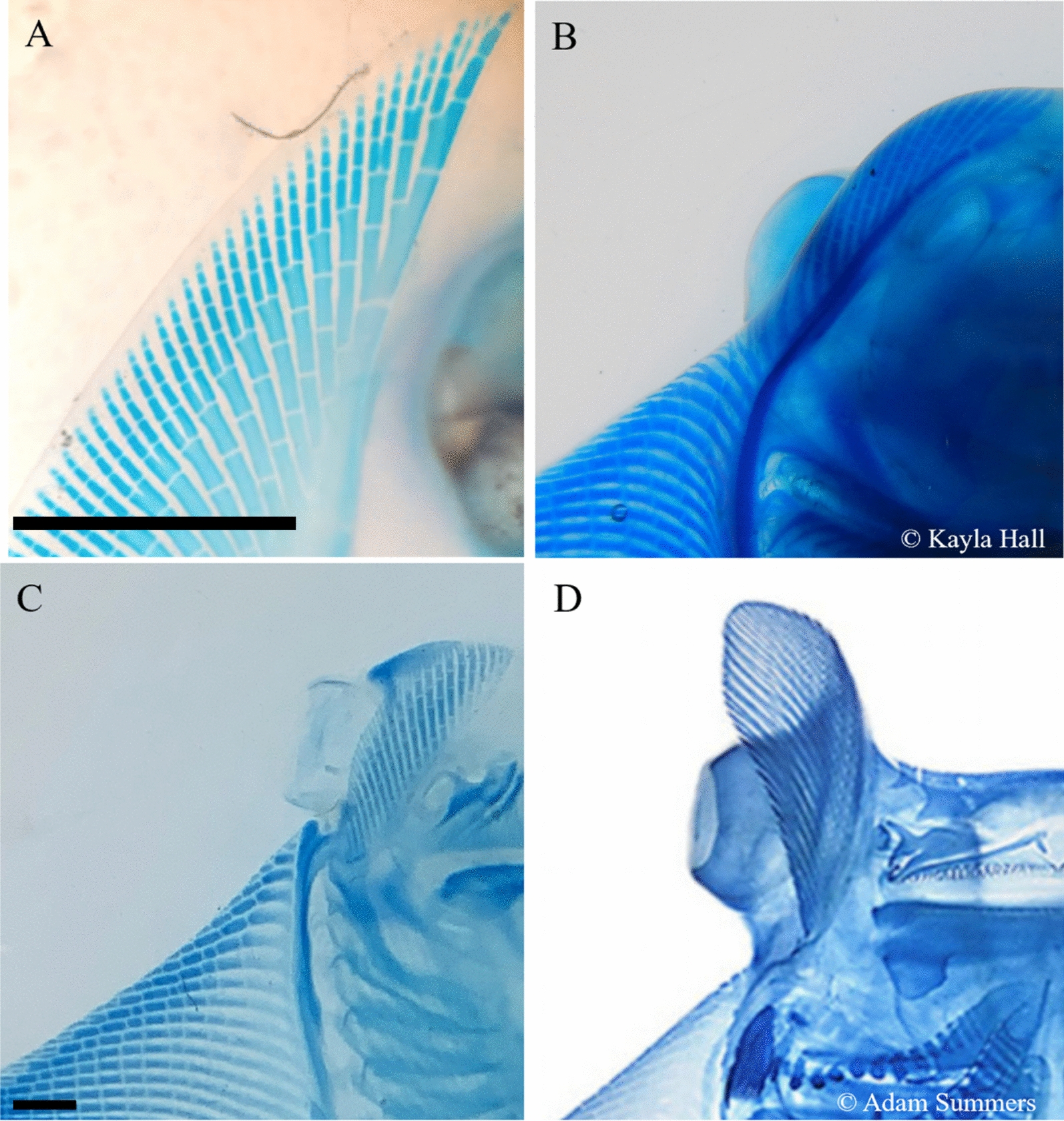
Variation in pectoral fin morphology between a little skate (**A**, no cephalic fins) and three myliobatid rays with cephalic fins (**B**–**D**) that evolved by splitting the pectoral fin into two distinct domains via interruption of fin ray outgrowth. **A**: Dorsal view of the anterior pectoral fin with a continuous margin of a little skate (*Leucoraja erinacea*). **B** Ventral view of the pectoral fin of a California bat ray (*Myliobatis californica,* © Kayla Hall) featuring the cephalic fin separated from the pectoral fin by a region of reduced fin ray outgrowth (which we refer to as the “notch region). **C**: Ventral view of the cephalic fin and pectoral fin of a cownose ray (*Rhinoptera bonasus*). **D** Ventral view of the cephalic fin and pectoral fin with the most extreme “notch” region of a *Mobula* ray (© Adam Summers). Scale bar on **A** and **C** represents 2.5 mm. Scale was not available for **B** and **D**

This morphological and functional domain splitting manifests as reduced fin ray outgrowth (Fig. 1), creating separate modules that can be optimized independently by natural selection. Therefore, functional separation of these two pectoral fin domains might be considered a key innovation [6] in myliobatid evolution. Subsequent modifications in cephalic fins include a novel tendon and muscle that facilitate independent operation [1]. In addition, several pectoral fin modifications arose that likely facilitated invasion of the pelagic environment and a novel mode of locomotion called oscillatory swimming, or “underwater flight” [7]. For example, anterior pectoral fin rays are thicker [8] and exhibit derived patterns of cross-bracing [9], the number of fin rays is asymmetric with redistribution towards the posterior [8], the center of mass is shifted [9], and the aspect ratio (the ratio of disk length to disk width) is high [10]—all features that improve swimming efficiency in pelagic environments. While the selective advantage of domain splitting in this context is apparent, the developmental mechanism associated with this morphological transformation has not been tested functionally.

Fins and limbs share the same underlying genetic repertoire during development, including maintenance of the apical ectodermal ridge (AER) by *Wnt3* (in mouse, [11]) or *Wnt3a* (in chick, [12]) expression around the perimeter of the fin/limb bud, driving distal outgrowth. During batoid pectoral fin expansion, the anterior-most domain adopts a “hook-like” morphology as the fins grow anterodistally. In myliobatid rays, this anterior domain represents the cephalic fin and becomes visibly delineated from the rest of the pectoral fin in a region where outgrowth is inhibited (referred to hereafter as the “notch”), splitting the fin into two domains [5]. While several studies have demonstrated that the expanded batoid pectoral fin is associated with a novel, anterior AER [13, 14], the purpose of this research is to functionally evaluate whether interruption of that AER is associated with pectoral fin remodeling and the evolution and development of cephalic fins via modulation of fin ray length.

Although manta rays are the most well-known taxa exhibiting cephalic fins and bear the most conspicuous example of this unique feature, all species are threatened and vulnerable to extinction. However, the cownose ray (*Rhinoptera bonasus*) is abundant off the United States east coast, and, as a member of the sister genus to *Mobula*, acts as a tractable proxy for manta ray evolution. Swenson et al. [5] characterized differential gene expression in the anterior pectoral fins of the cownose ray compared to the little skate (*Leucoraja erinacea*; a related taxon lacking cephalic fins). Candidate genes were identified based on differential expression in anterior pectoral fins of the little skate or the cownose ray, but not both (i.e., to identify differences in pectoral fin development between these taxa). This process culminated in a short list of candidates which had known functions in fin/limb outgrowth and development pathways. *Dkk1* and *Vsnl1* are significantly upregulated in the anterior pectoral fin of the cownose ray (but not little skate), while *Msx1* and *Lhx2* are significantly upregulated in the anterior pectoral fin of in the little skate (but not cownose ray, [5]). Interestingly, *Dkk1* is a known inhibitor of the canonical β-catenin-dependent Wnt signaling pathway during AER outgrowth, and unlike *Vsnl1*, it is an extracellular signaling molecule, making it easy to introduce ectopically. The factors together make *Dkk1* the highest priority candidate gene.

Given that AER maintenance drives fin/limb outgrowth and β-catenin-dependent Wnt signaling is necessary for AER initiation and maintenance [15], disruption of this signaling pathway is a plausible model for interruption of the AER. This disruption can be identified through changes in transcription of Wnt signaling target genes, such as *Axin2* and *Fgf8* [16–18]. For example, a well-characterized method of interrupting β-catenin-dependent Wnt signaling is competitive inhibition by *Dkk1*, which blocks the binding sites for Wnt ligands [19]. *Axin2* is a direct target of the Wnt signaling cascade and a tractable marker of active β-catenin-dependent Wnt signaling [20–22]. In addition, in situ hybridization (ISH) has revealed that *Axin2* expression during embryogenesis has significant overlap with *Wnt3/Wnt3a* in the limb AER of mouse, chicken, and axolotl [22–25], validating its use as an AER marker.

This study aims to functionally validate the role of *Dkk1* in pectoral fin domain splitting of manta rays and their relatives by introducing ectopic DKK1 protein to the anterior pectoral fins of little skate embryos, phenotypically “turning a skate into a manta ray.” The effects of this introduction are visualized in two different ways: morphologically, using clear and stain to examine the resulting pectoral fin phenotypes, and genetically, using ISH of *Axin2* as a readout for interruption of *Wnt3* signaling by ectopic expression of *Dkk1*. In addition, we investigate wildtype expression of the following candidate genes using ISH: *Dkk1* in the cownose ray, and *Msx1* and *Lhx2* in the little skate. This combined approach allows us to compare introduced DKK1 and its resulting phenotype in a non-myliobatid taxon while demonstrating that *Dkk1* is expressed in cownose ray during periods of pectoral fin remodeling.

## Materials and methods

### Animal husbandry and staging

Little skate embryos were obtained from Marine Biology Laboratory (Woods Hole, MA) at stages 28–31 (following [26, 27]), and preserved for gene expression analyses in 4% paraformaldehyde (PFA) for 48 h before moving to 100% methanol for storage at −20 °C. In addition, we were able to obtain three cownose ray embryos at stage 3 (homologous to little skate stage 31; embryos acquired from and staged according to Ref. [5]) that were fixed in 4% paraformaldehyde (PFA), and stored at −80 °C in methanol, for use in ISH.

Live embryos were kept at 15 °C in reconstituted Instant Ocean with 12h light-dark cycles. For functional assays, embryos were removed from eggs cases and transferred into 118ml clear plastic containers, which were floated in the tanks. Each container was modified with holes to allow for water exchange while containing and protecting the embryo, mimicking the environment of the egg cases.

### Probe synthesis

RNA was extracted from tissues preserved in RNA*later*® using the QIAGEN RNeasy® Micro Kit and converted to cDNA using the Applied Biosystems™ High-Capacity cDNA Reverse Transcription Kit. Probe sequences for ISH were obtained from public databases when available, or by designing degenerate primers based on multiple sequence alignment. Probes were synthesized from constructs targeting *Dkk1*, *Msx1*, *Lhx2*, and *Axin2* using pGEM-T Easy Vector System (Promega) and following the protocol of Wilkinson [28].

### ISH

Whole-mount mRNA ISH ([28] with modifications from [14]) was performed on little skate embryos (stages 28–31, following [26, 27]) to detect *Axin2*, *Wnt3*, *Msx1*, and *Lhx2* expression and on cownose ray embryos (stage 3, homologous to little skate stage 31, following [5]) to detect *Dkk1* expression. *Dkk1* expression in little skate embryos could not be detected using ISH. Post-ISH embryos were re-fixed in 4% PFA, photographed in 100% glycerol, and stored at 4 °C.

### Bead implantations

Bead implantations were performed according to published methods [13, 29] and guidance from K.L. O’Shaughnessy (personal communication) with the following modifications: Affigel-Blue (Bio-Rad, 1537302) beads were soaked overnight at 4 °C in DKK1 protein (mouse origin; R&D Systems, 5897-DK-010) reconstituted at 100 μg/mL in PBS containing 0.1% bovine serum albumin (BSA) or, as a control, in PBS containing 0.1% BSA. Embryos at late stage 30 and early stage 31 were removed from their egg cases and anesthetized using MS-222 following Westerfield [30]. Following anesthesia, a bead soaked in DKK1 was implanted in the anterior margin of the left pectoral fin on the ventral side. A control bead was then implanted in the same manner on the right pectoral fin. Implantations were performed using tungsten needles (0.001 mm tip diameter; Fine Science Tools, 10130-10). Embryos were returned to containers and tanks and incubated for 6, 12, 24, 36, and 72 h for use in *Axin2* ISH, or for 4 weeks for phenotypic analysis via Alcian blue staining following Gillis and Shubin [31].

## Results

### DKK1 interrupts fin ray outgrowth and development in the little skate pectoral fin, recapitulating the myliobatid phenotype

**Fig. 2:**
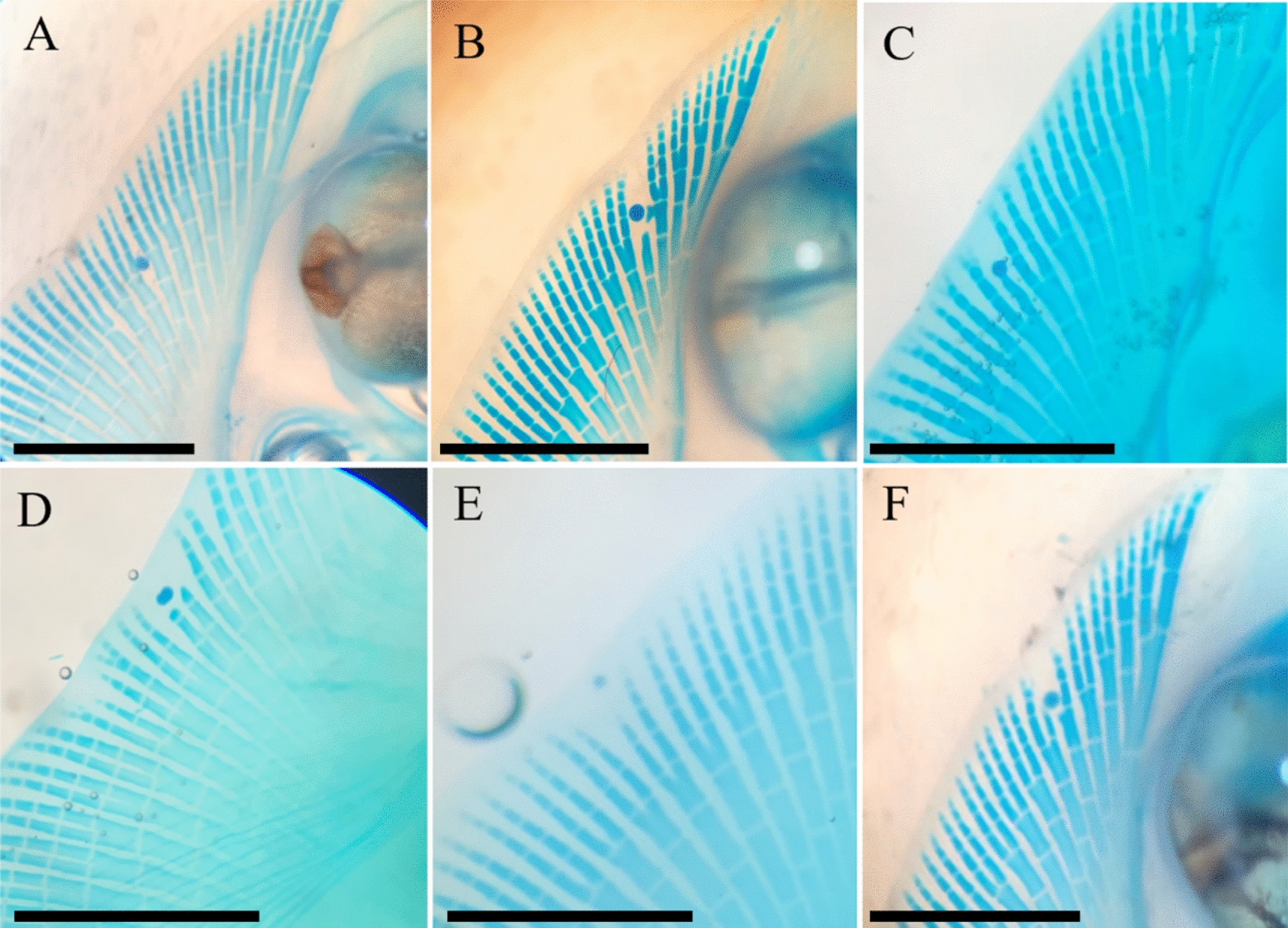
DKK1 bead implantation interrupts fin ray outgrowth in the little skate. Compare the control bead (**A**) vs. DKK1-soaked beads (**B**–**F**). Note the reduced fin ray outgrowth in 100% embryos implanted with a DKK1-soaked bead (*n* = 21), which is not observed with the control bead. The truncated fin rays observed in association with DKK1 bead implantations (**B**–**F**) resemble the shorter fin rays observed in the “notch” mirroring the early stages of pectoral fin domain splitting in the myliobatid phenotype (Fig. 1). See text for explanation of why the phenotype could not be driven to completion due to limited activity of the ectopic DKK1 protein. Pictures show dorsal views of the anterior pectoral fins of six different little skate embryos that have been incubated for 4 weeks after bead implantation. Scale bar represents 2.5 mm

We implanted DKK1-soaked beads on the left pectoral fin and PBS-soaked control beads on the right pectoral fin in 21 little skate embryos (stgs. 30–31) and incubated for 28 days. While the ectopic DKK1 protein was spatially limited and exhibited diminished effect by 3 days, this incubation period allowed the embryos to progress to stage 32, at which point the distal elements of the fin rays have mineralized and can be stained. We used clear and stain protocols (following [31]; diaphanization) to visualize effects of introduced DKK1 on skeletal phenotypes and found clear evidence for interruption of fin ray outgrowth with reduced fin ray length near the bead (Fig. 2). We found fin ray truncation in 100% of embryos with implanted DKK1 beads, and effects were constrained to the distal elements that developed after bead implantation and during the incubation period. We used two metrics to document phenotypic effects: number of fin rays affected and number of segments absent/perturbed. All DKK1 beads resulted in truncation of adjacent fin rays in a small radius around the bead (1–3 rays, *n* = 21, $\overline{x }$= 1.5). Truncation was also quantified by the number of absent segments, which varied from 2 to 11 (*n* = 21, $\overline{x }$= 4.38). Of the control beads that were implanted, 62% (13 of 21) exhibited no developmental effects; therefore, most control bead implantations exhibited normal growth and development around the bead. However, 38% (8 of 21) of embryos implanted with control beads exhibited slight disruption of fin ray development due to mechanical damage during bead implantation, which was challenging. Taking this into consideration, we performed a *t*-test (two-tailed, equal variance) indicating the differences for both number of truncated rays and total absent segments were highly significant with *p* values of 4.7901 × 10^–8^ and 5.27697 × 10^–9^, respectively, as was the number of embryos effected with a *p* value of 7.0116 × 10^–6^.

### DKK1 interrupts *Wnt3* signaling/*Axin2* expression in the pectoral fin AER of the little skate

**Fig. 3:**
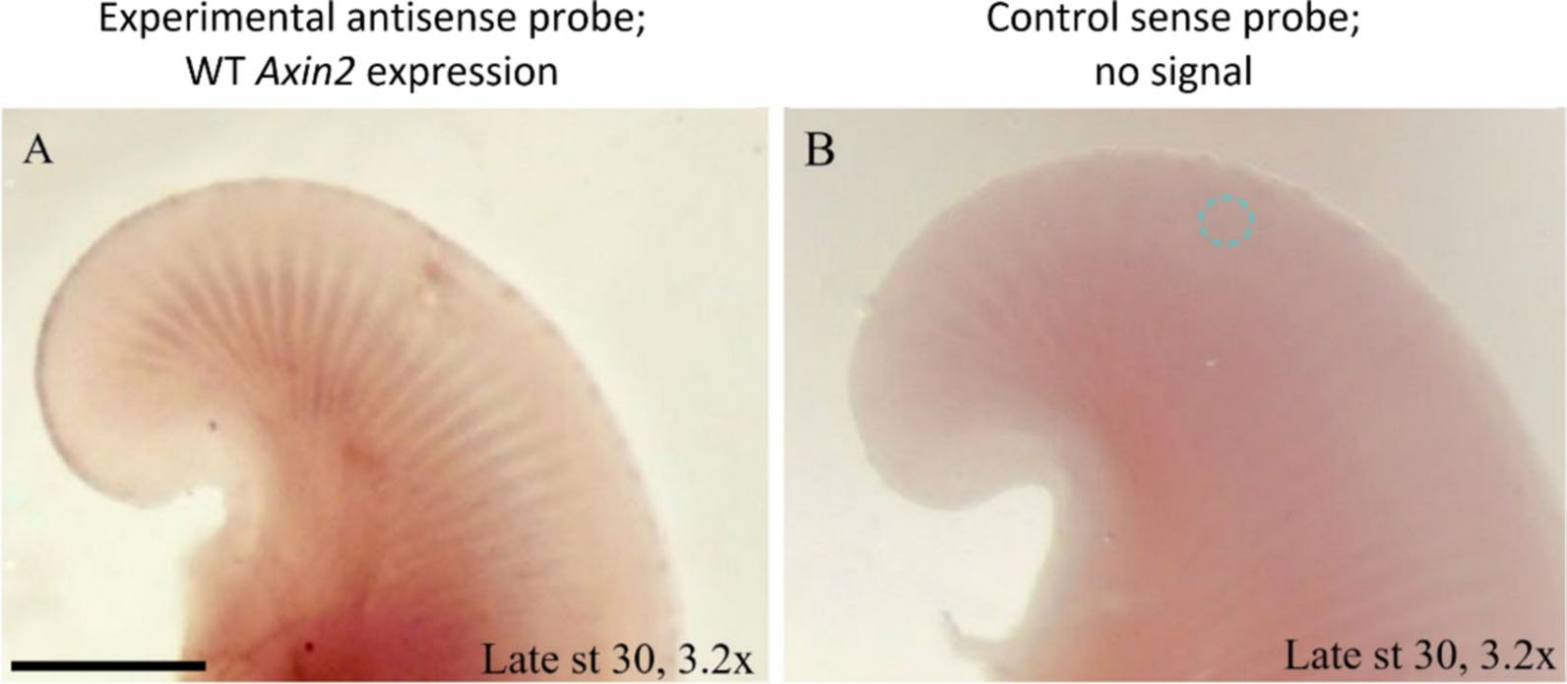
Wildtype *Axin2* is expressed in the little skate anterior pectoral fin AER at stage 30 (**A**) compared to a fin treated with the control probe (**B**), demonstrating no signal. *Axin2* is expressed throughout the AER in a fine line and trails off in a gradient posteriorly along the fin at this stage. The blue dotted circle represents the location of a PBS-soaked control bead, which has been edited out for clarity. Scale bar represents 1 mm

**Fig. 4:**
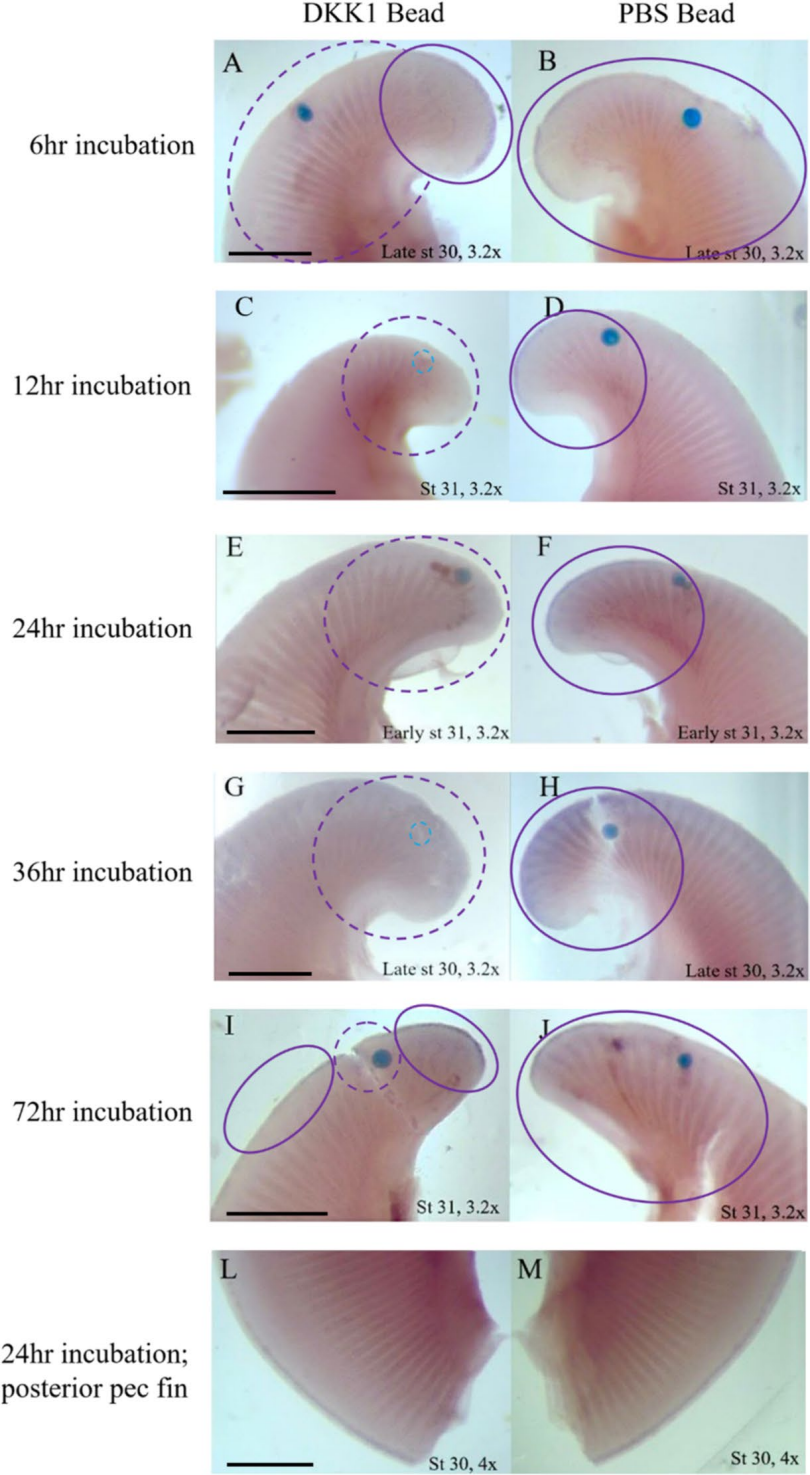
Ectopic DKK1 interrupts AER maintenance/*Axin2* expression in the little skate pectoral fin for up to 72 h. Compare left column with interrupted *Axin2* expression near the *Dkk1* soaked bead with the right column that shows stage-specific native expression with implantation of a control bead. *Axin2* expression is depicted in the anterior pectoral fins of little skate embryos at stages 30 (**A**, **B**; **G**, **H**) and 31 (**C**–**F**; **I**, **J**) of development after implantation of agarose beads soaked in DKK1 protein in the left fin (left column) and PBS in the right fin (right column) with the following incubation times: 6 h (**A**, **B**), 12 h (**C**, **D**), 24 h (**E**, **F**), 36 h (**G**, **H**) and 72 h (**I**, **J**). Comparisons between the DKK1 (left pectoral fin) and PBS control (right pectoral fin) beads illustrate treatment effects from implantations in the same individual. In some individuals (**C**, **D** and **G**, **H**), beads came out during the ISH process. Former location of beads is visible and denoted by blue dashed circles. Solid purple circles denote regions with *Axin2* expression and dashed purple circles denote regions where *Axin2* expression is expected but has been interrupted. For all incubation times, PBS beads had no effect on *Axin2* expression. After 6 h, interruption of AER-associated *Axin2* expression in the DKK1 treatment is clearly visible near the bead (**A**, **B**). After 12, 24, and 36 h incubation times, *Axin2* expression in the anterior pectoral fins was completely interrupted. After 72 h of incubation (**I**, **J**), the effect of DKK1 inhibition on *Axin2* is diminished but still visible near the bead, suggesting that the temporal scale of DKK1 protein activity is approximately 1–3 days. *Axin2* expression in the posterior pectoral fins (**L**, **M**) was not affected in any individual regardless of treatment or incubation time, suggesting a spatial scale of approximately 1 cm from the implanted bead. Posterior pectoral fins at 24 hpi are depicted as this is when DKK1 reaches peak penetrance. Sense probe (see **B**) showed no staining. Since embryos with different incubation times were at different developmental stages, the expected expression domain of *Axin2* varies accordingly. Scale bar represents 1 mm

We used *Axin2* expression as a readout for AER outgrowth. In the little skate, wildtype expression at stage 30 occurs in the anterior and posterior third of the pectoral fin at the distal ridge of the AER, marking the regions with continued AER outgrowth (Fig. 3A), compared to the RNA sense probe (i.e., ISH control, Fig. 3B). At stage 31, expression is retained in in the anterior quarter of the fin as it continues to grow anteriorly, but diminishes to a much smaller region in the posterior fin. To test the spatial and temporal scale of DKK1 activity, we performed a titration experiment, incubating for five different time intervals post-implantation. Beads were implanted in embryos at stages 30 (Fig. 4A–B; G–H) and 31 (Fig. 4C–F; I–J) alike, and although wildtype *Axin2* expression differs slightly between these stages, the downstream impacts of DKK1 on expression are clear regardless of stage (Fig. 4).

**Fig. 5:**
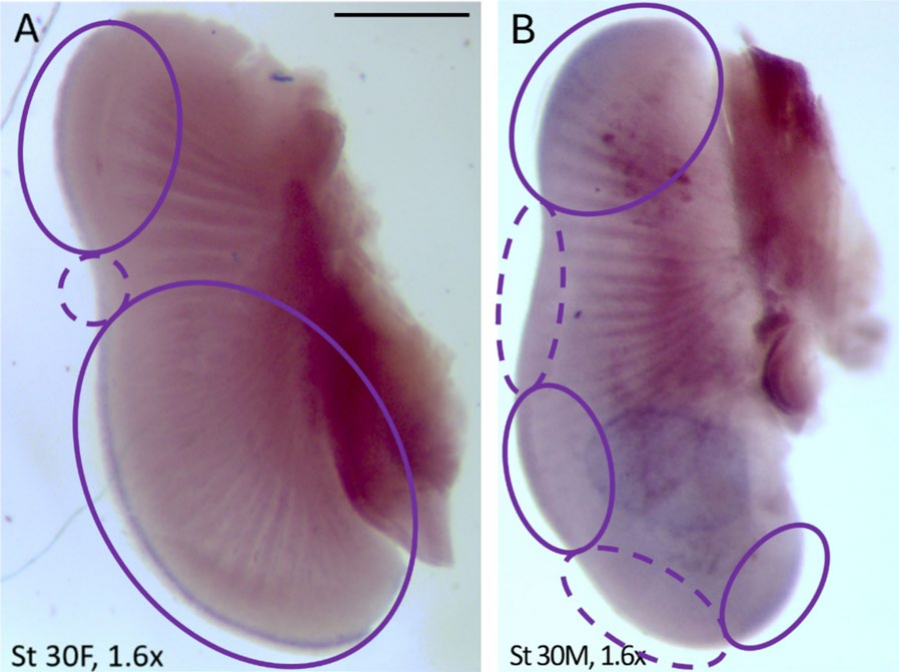
*Axin2* is expressed in the AERs of little skate pelvic fins at stage 30. **A** *Axin2* expression in the pelvic fin of a female skate at stage 30. Two expression domains can be observed: one in the crura and one in the posterior lobe of the fin. **B** *Axin2* expression in the pelvic fin of a male skate at stage 30. Solid purple circles denote regions in with *Axin2* expression and dashed purple circles denote regions where *Axin2* expression is expected but has been interrupted. Three expression domains can be observed: one in the crura, one in the posterior lobe of the fin, and one in the clasper. Sense probe showed no signal. Scale bar represents 0.5 mm

*Axin2* expression was interrupted in a small region around a DKK1-soaked bead implanted in the left anterior pectoral fin AER of all five treated embryos (Fig. 4 left column), while a PBS-soaked control bead implanted on the right side of the same embryo indicates wildtype expression with no interruption from bead implantation (Fig. 4 right column). Posterior pectoral fin AER expression was not altered in fins that received DKK1 beads in the anterior, indicating a local effect of the DKK1 protein. The degree of *Axin2* inhibition was modulated by the length of incubation following bead implantation. At 6 h post-implantation (hpi), the effect was already observable (Fig. 4A, B). In the DKK1 bead treated fin, *Axin2* expression was interrupted in a noticeable halo around the bead in the anterior pectoral fin. After 12 hpi, the difference was more dramatic, with the fins treated with DKK1 beads exhibiting complete absence of *Axin2* expression (Fig. 5C-D). This effect remained constant at 24 (Fig. 5E, F) and 36 (Fig. 5G, H) hpi. However, after 72 hpi (Fig. 5I, J), *Axin2* expression is restricted to a small halo around the DKK1 bead, likely due to diminished activity of the DKK1 protein after 3 days. To our knowledge, this is the first documented experiment to evaluate and titrate temporal and spatial scales of DKK1 activity, illustrating interruption on β-catenin-dependent Wnt signaling in the developing fin/limb. In summary, this analysis indicates that DKK1 remains active for 36–72 h, with peak penetrance observed at 24 hpi.

Finally, *Axin2* was also detected in the AERs of the pelvic fins (Fig. 5). At stage 30 in females (Fig. 5A), posterior expression is continuous, whereas in males (Fig. 5B), expression is interrupted at the “notch” of the developing clasper, a pattern which persists in stage 31 (Fig. 5C) as the claspers become more morphologically differentiated from the posterior lobe of the clasper. At both stages 30 and 31, expression is also discontinuous between the crura and posterior lobe of the pelvic fin in both sexes.

### *Dkk1* expression in the cownose ray AER is associated with pectoral fin remodeling via reduced fin ray length in anterior (including cephalic fins) and posterior pectoral fins

**Fig. 6:**
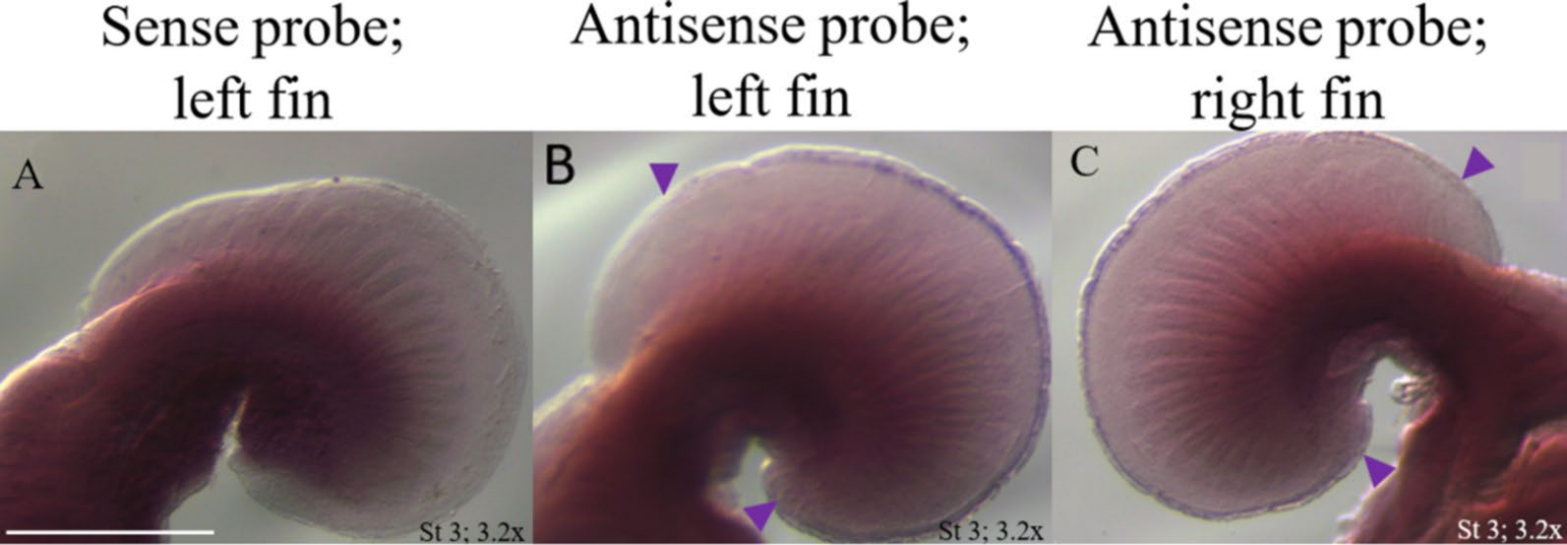
*Dkk1* expression modifies AER outgrowth during pectoral fin development. Here, we demonstrate *Dkk1* expression in cownose ray cephalic fins at stage 3 (stage 31 in skates), the only stage available for this experiment, which is after development of the “notch”. **A** No staining with the *Dkk1* control sense probe. **B**, **C** Expression in the distal ridge of the AER indicated by purple staining with the *Dkk1* antisense experimental probe on the left side (**B**), and right side (**C**). Purple arrows denote regions where *Dkk1* expression occurs. While *Dkk1* was expressed in the “notch” region at an earlier stage (based on [5]), it is still expressed in the cephalic fin AER where fin ray length is somewhat reduced. Scale bar represents 2.5 mm

*Dkk1* expression was detected in the developing cephalic fins (i.e., anterior pectoral fins) of a cownose ray embryo at stage 3 (homologous to little skate stage 31; Fig. 6), consistent with the findings of Swenson et al. [5], as well as posterior pectoral fins. These represent two regions where the fin rays are reduced by fine-tuning AER outgrowth by DKK1 (as observed above). It is worth noting that, at stage 3 (stage 31 in skates), the split in the pectoral fins delineating the cephalic fins had already been completed, therefore we were unable to visualize *Dkk1* expression in the region of pectoral fin domain splitting (i.e., the notch) because we did not have embryos at the developmental stage when the split initiates. We would expect that ISH of a cownose ray embryo prior to notch formation would detect maximum *Dkk1* expression localized to the region where the notch forms, in association with AER interruption. Unfortunately, attempts to obtain additional embryos at stages 1–2 (stages 29–30 in skates) were unsuccessful. While Swenson et al. [5] demonstrated upregulation of *Dkk1* in the anterior pectoral fin of the cownose ray (including the notch), they found no differential expression in the little skate anterior pectoral fin.

### *Lhx2 *and *Msx1* expression are associated with the three novel AERs in skates and exhibit interruption at the notch

**Fig. 7:**
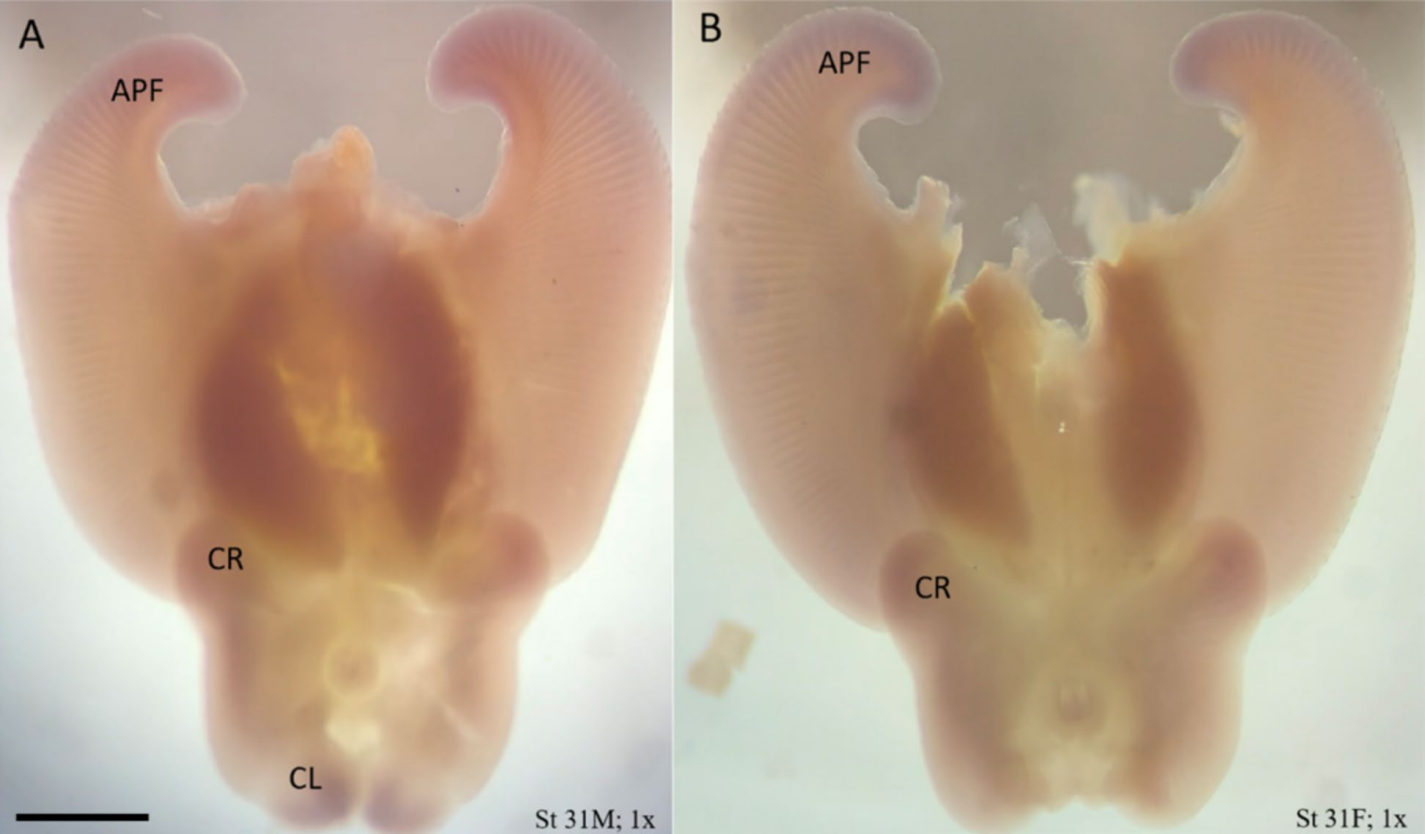
Expression of *Lhx2* in stage 31 little skate embryos is associated with anterior expansion and clasper development. **A** The ventral view of a male little skate embryo at stage 31. **B** The ventral view of a female little skate embryo at stage 31. Broadly diffuse expression underlying the AER is present in the anterior pectoral fins, the crura, and the claspers [male only, (**A**); note that claspers do not occur in females (**B**)]. Heads, tails, and body tissue have been dissected to improve visibility of expression in fins. APF: anterior pectoral fin; CR: crura; CL: clasper. Sense probe (SI Fig. 2) showed no staining. Scale bar represents 5 mm

*Lhx2* and *Msx1* were found to be enriched in the anterior pectoral fin in the little skate (relative to the to the posterior pectoral fin, [5]), therefore, we performed ISH to illustrate fine-scale spatial mapping of expression at stage 31. We found broadly diffuse expression of *Lhx2* (Fig. 7) in the underlying tissue of all three (previously described) novel AERs in the little skate (anterior pectoral fin, [13]; crura, [14]; and clasper, [29]), suggesting a role in AER maintenance and developmental outgrowth via mesenchymal induction/interaction. Mesodermally expressed *Lhx2* has previously been implicated in maintaining the FGF-to-SHH regulatory loop, which mediates the AER (in the posterior zone of polarizing activity, ZPA) in mouse limb development [32]. Interestingly, we did not see *Lhx2* expression in the posterior pectoral fin (i.e., ZPA region). Furthermore, *Shh* expression is not associated with the anterior pectoral fin or crura at stage 31 [13, 29] where we see *Lhx2* expression in skate paired fins, indicating a role for *Lhx2* in maintaining the AER that is independent of the ZPA organizing center. Notably, there is an interruption of *Lhx2* expression in the region of the notch defining clasper development that is consistent with the relative increased expression in skate compared to myliobatid rays (see [5]).

**Fig. 8:**
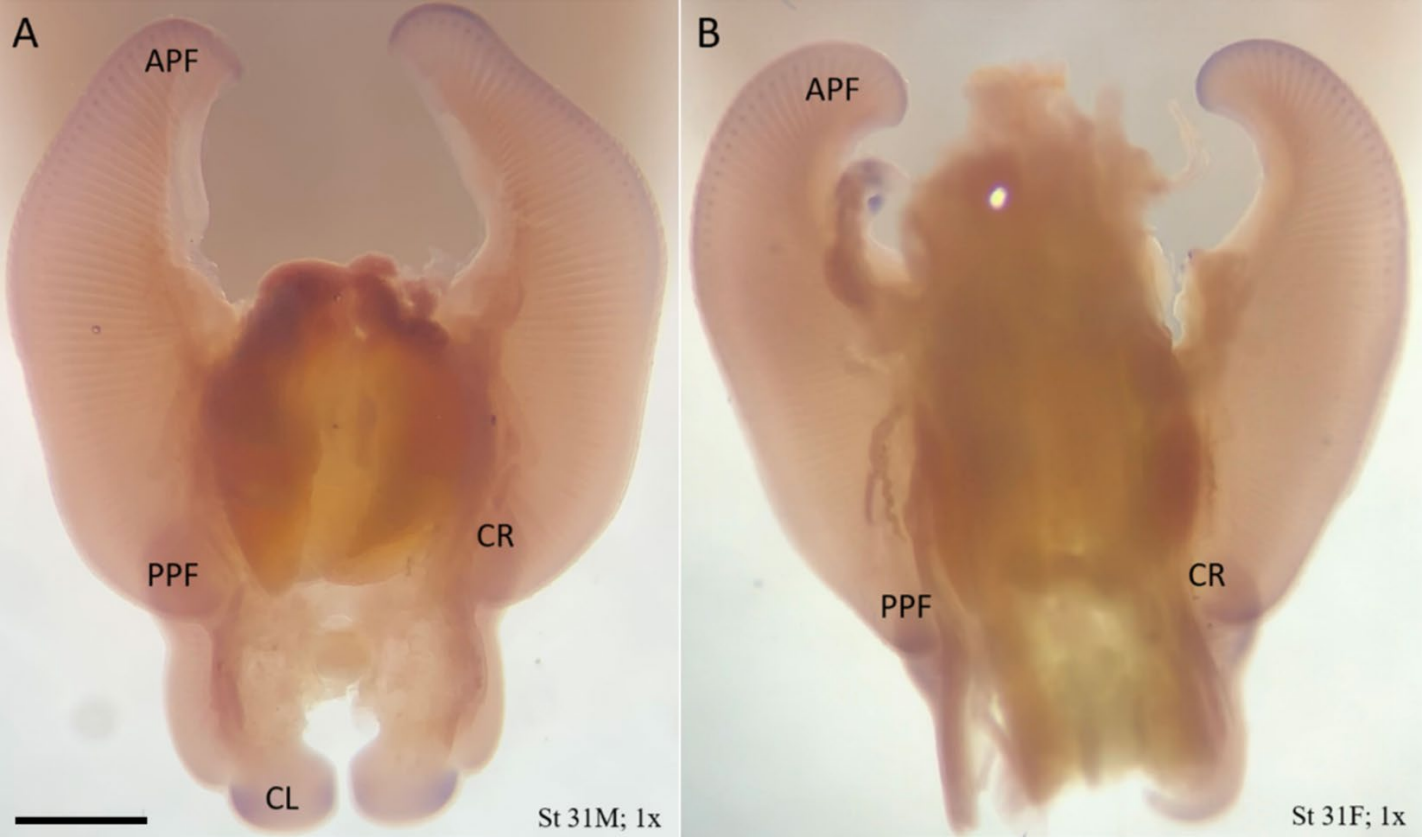
Expression of *Msx1* in stage 31 little skate embryos is associated with the AER, fin ray outgrowth, and clasper development. **A** The ventral view of a little skate embryo at stage 31 in a male (**A**), and female (**B**). Sharply defined expression at the AER in the anterior and posterior pectoral fins, the crura, posterior pelvic fins, and more broadly diffuse expression in the medial and lateral domains of the claspers in the male (**A**) that may be associated with the rolled morphology of claspers. Expression is sharpest and most intense in the anterior pectoral fins. The expression domain in the claspers is more broad and is associated with regions that eventually will curl to give shape to the clasper. Tiny dots of expression can also be seen associated with the tips of fin rays in the anterior pectoral fins. Heads, tails, and body tissue have been dissected to improve visibility of expression. APF: anterior pectoral fin; PPF: posterior pectoral fin; CR: crura; CL: clasper. Sense probe (SI Fig. 2) showed no staining. Scale bar represents 5 mm

**Fig. 9:**
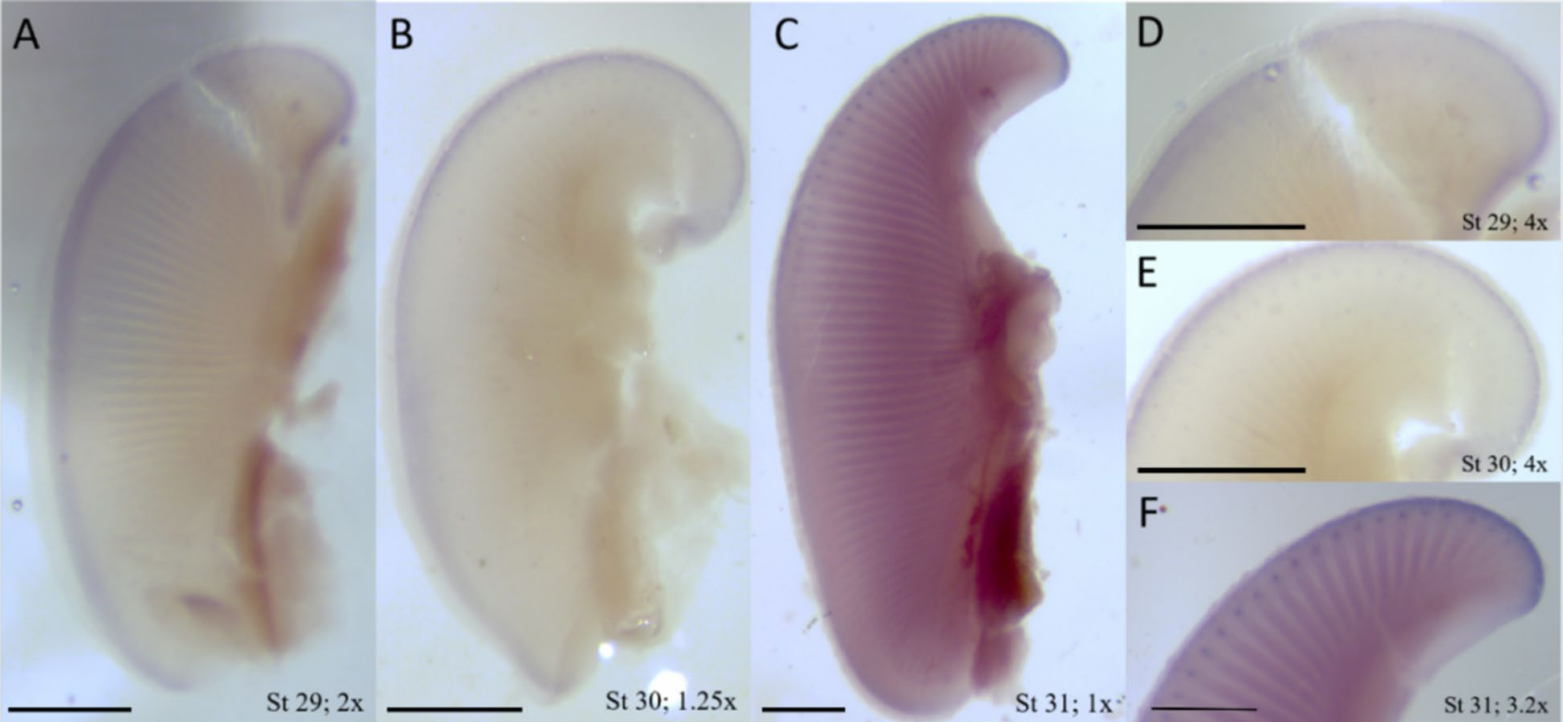
Pectoral fin expression of *Msx1* in little skate embryos at stages 29–31 is associated with the AER and anterior fin ray outgrowth. **A** The pectoral fin of a little skate at stage 29. *Msx1* expression is consistent around the perimeter of the entire fin. **B** The pectoral fin of a little skate at stage 30. *Msx1* expression can still be detected around the entire fin, but it strongest in the anterior. **C** The pectoral fin of a little skate at stage 31. *Msx1* expression is strongest in the anterior and is still expressed in the posterior, but expression weakens mid-fin. **D** A close-up of the anterior pectoral fin of a little skate at stage 29. Dots of expression associated with fin rays are extremely faint. **E** A close-up of the anterior pectoral fin of a little skate at stage 30. Dots of expression associated with fin rays are becoming more apparent. **F** A close-up of the anterior pectoral fin of a little skate at stage 31. Dots of expression associated with fin rays are extremely prominent. Sense probe (SI Fig. 2) showed no staining. Scale bar represents 1 mm

Expression of *Msx1* is sharply defined at the anterior AER of the pectoral fin of the little skate at stage 31 (Fig. 8) with no expression in mid pectoral fin, and relatively subtle expression in the posterior pectoral fin AER (Fig. 9C). It is also expressed in the anterior AER of pelvic fins, corresponding to the region of crura outgrowth (Fig. 8). *Msx1* exhibits a more broadly diffuse expression domain in the underlying mesenchymal tissue of the developing claspers of males (similar to and overlapping with *Lhx2*) but enhanced medially and laterally, which may be associated with the rolled morphology of claspers. Finally, dots of *Msx1* expression are associated with the distal tip of the developing anterior fin rays (24–28 rays, $\overline{x }$=26.3, Fig. 9F) that is nearly undetectable at stage 29 (Fig. 9D) and somewhat apparent at stage 30 (Fig. 9E) with maximum expression in the dots at stage 31 during peak *Msx1* expression at the perimeter of the fin (Fig. 9A, B). Interestingly, we demonstrate interruption of *Msx1* at the notch defining clasper development at stage 31, similar to what was observed with *Lhx2*.

## Discussion

### The role of *Dkk1* in domain splitting via inhibition of fin ray outgrowth

Introduction of ectopic DKK1 interrupted fin ray development, resulting in a morphology of shorter fin rays with missing segments. The spatial scale of the impact of DKK1 bead implantation on fin ray development spanned 1–3 fin rays. We demonstrate that the DKK1 protein effect attenuates after 72 hpi, based on the results of the *Axin2* titration (Fig. 4). Within this time frame of demonstrated DKK1 activity, we were able to observe phenotypes consistent with domain splitting as observed in cephalic fins of myliobatid rays (Fig. 2A–C). Pectoral fin remodeling depends on alterations in (i.e., fine-tuning) the duration, location, and dosage of gene expression associated with fin ray outgrowth—such as *Dkk1*, which we demonstrate functions as an outgrowth inhibitor. In myliobatid rays, we expect *Dkk1* expression to be associated with areas of reduced fin ray length (i.e., the notch region and anterior/posterior pectoral fin). This is supported by the observation that *Dkk1* inhibits fin ray outgrowth in teleosts. Aman et al. [33] found that when *Dkk1* expression is induced in developing transgenic zebrafish (*Danio rerio*), outgrowth of the pectoral fin rays is significantly diminished, while pelvic fin rays (which develop after pectoral fins) do not develop at all. Actinotrichia are the first skeletal elements to form during fin ray development and are homologous to ceratotrichia, which form the fin rays in cartilaginous fishes [34, 35]. These effects of introduced DKK1 support the common role of *Dkk1* in shaping fin ray elements originated in the common ancestor of jawed vertebrates.

### Exposure to ectopic DKK1 inhibits β-catenin-dependent Wnt signaling in pectoral fins and interrupts outgrowth at the AER

To functionally validate the role of DKK1 in disrupting the AER via inhibition of Wnt signaling, we visualized expression of a gene that both serves as a visual AER marker and is a direct downstream target of Wnt signaling. *Axin2* has been widely used as a readout for Wnt signaling in quantitative experiments involving RT-PCR (e.g., [21, 36, 37]) and ISH experiments (e.g., [21, 24, 38]). Ellwanger et al. [39] included *Axin2* as an AER marker in addition to *Fgf8* when knocking down inhibitors of Wnt signaling *Kremen1*, *Kremen2*, and *Dkk1*. When these Wnt signaling inhibitors were removed, β-catenin-dependent Wnt signaling increased, expanding the expression domain of AER marker genes, including *Axin2*. This illustrates that removal of *Dkk1* inhibition (thus, increased DKK1 activity) directly leads to an increase in *Axin2* transcription in the AER. While the negative relationship between DKK1 and *Axin2* has been demonstrated quantitatively (e.g., [36, 37]), to our knowledge, our data represent the first experiment to illustrate (via ISH) that ectopic DKK1 leads to downregulation of *Axin2*. *Fgf8* is typically used as an AER marker for such experiments [40–45] because, as demonstrated herein, the signal for *Axin2* in the AER is not particularly strong [24, 45, 46]. However, for the purposes of this study, *Axin2* is the best choice for an anterior AER marker because Nakamura et al. [13] demonstrated that *Fgf8* is only expressed in the posterior pectoral fins of the little skate.

Fins that received DKK1-soaked beads exhibited interruption of *Axin2* expression. Fins exhibiting wildtype phenotypes (i.e., with PBS-soaked control beads or without bead implantation) demonstrate *Axin2* expression in the AER (based on known *Wnt3* expression [13]), consistent with the detection of *Axin2* expression in tetrapod AERs (chicken [24], mouse [38], axolotl [25]) and in the developing paired fins of teleosts (zebrafish, [40, 41]). The degree of interruption varied according to the length of incubation, with peak interruption at 24 hpi, which was maintained through at least 36 hpi. At 72 hpi, *Axin2* expression and AER function is nearly restored throughout the anterior pectoral fin, aside from a small area directly adjacent to the bead. This area corresponds to the region of fin ray interruption seen in the embryos incubated for 4 weeks (spanning stages 30–32). Although mechanical damage interrupted fin ray growth in some embryos that received PBS beads, the consistent disruption of *Axin2* expression associated with DKK1 beads and the wildtype expression associated with PBS beads indicates the specific effect of DKK1. Sustained expression of DKK1 in myliobatid rays could easily explain the domain splitting phenotype observed in the notch region in cownose ray embryos. Interestingly, Mukhopadhyay et al. [47] found that ectopic expression of *Dkk1* in chick limb buds also caused the AER to split into two domains, consistent with a putative role for *Dkk1* in paired fin/limb domain splitting in myliobatids, verifying that inhibition of *Wnt3* signaling activity by DKK1 causes phenotypes in the AER of tetrapods, as well as novel morphologies in cartilaginous fishes.

### A model for paired fin remodeling that invokes *Dkk1* as the “brake pedal” and Wnt3 as the “gas pedal” that shape distal fin morphologies via inhibition and maintenance of the AER

We propose a model for distal limb outgrowth in which *Dkk1* acts as a “brake pedal,” with the Wnt-dependent AER acting as the corresponding “gas pedal.” If the AER in this region is no longer active, *Dkk1* expression would not be necessary to inhibit growth. In short, we only expect to see use of the “brake pedal” when the “gas pedal” is also active. As such, we detected *Dkk1* expression in the AER of developing cephalic fins of stage 3 cownose ray embryos (stage 31 in skates), after domain splitting had already occurred, and the notch was fully formed. In these embryos, expression was not detected in the notch region, likely due to a lack of outgrowth in this area at this stage.

Further support for this model is the fact that *Dkk1* expression could also be detected in the posterior-most region of cownose ray pectoral fins and, weakly, in the pelvic fins (SI Fig. 4). We would expect that, prior to notch formation, when *Wnt3* is expressed around the entire perimeter of the fin, *Dkk1* is strongly expressed in the notch region, inhibiting AER-driven outgrowth. This concentrated notch expression would likely persist until the cephalic fins are defined and in regions with fine-tuning of fin ray outgrowth, as observed in the distinctive wing-like shape of myliobatids. While *Dkk1* is differentially expressed in anterior pectoral fin of cownose ray, consistent with domain splitting, there is non-differential expression in anterior and posterior pectoral fin of the little skate at stage 31 [5] consistent with A/P symmetry in skates. Surprisingly, we were unable to detect *Dkk1* expression in little skate at stages 29–31 via ISH, although expression in the pectoral fins has been previously confirmed via RNA-seq [5] and *Dkk1* sequences were successfully amplified from cDNA constructed from RNA extractions performed on little skate pectoral and pelvic fins at all three stages.

AER maintenance is dependent on *Msx* expression. *Msx1* is a homeobox gene expressed ubiquitously throughout the vertebrate fin/limb bud beginning in the earliest stages of development. Along with its paralog *Msx2, Msx1* is expressed in both the ectoderm and in the underlying mesenchyme of the limb bud. In the ectoderm, *Msx1* and *Msx2* are critical for AER maturation. In mouse embryos lacking these genes, limbs are truncated with digit malformations and the anterior AER fails to mature [48]. In mesenchymal tissue, *Msx1* and *Msx2* are involved in both BMP and Sonic hedgehog (SHH) signaling and function to modulate digit number and identity. In conditional knockout experiments in which *Msx1* and *Msx2* lose function only in the limb bud mesenchyme (not ectodermal AER, [44]), AER initiation and maintenance proceeds as normal, but misexpression of genes involved in the BMP and *Shh* signaling pathways lead to severe defects of the digits. Notably, the knockout resulted in misexpression of *Hand2* in the anterior portion of the limb bud—a gene that is enriched in both the cephalic fin and clasper of developing cownose ray embryos, two regions of domain splitting [13, 29]. This misexpression of genes creates an unusual polydactyl phenotype in which ectopic digits develop anteriorly and the typical anterior-most digits are highly truncated or even absent [49], resembling the distal modifications to myliobatid pectoral fin rays.

Interestingly, there is evidence supporting deep homology of fin rays and tetrapod digits, which share a distal patterning mechanism driven by *5’ Hox* activity [50], expression of the Shh/LIM/Gremlin/Fgf transcriptional network [51], and distal proliferation controlled by the *Shh/Gli3* gene regulatory network [52]. As a result of the teleost-specific genome duplication, zebrafish have two copies of *Msx1*: *msxB* and *msxE*, with *msxB* being the conserved copy with the sequence most similar to *Msx1* in tetrapods. Zebrafish *msxB* is expressed throughout the AER of the paired fin buds and at the tips of regenerated fin rays post-amputation [53]. Fin and limb regeneration has long been considered a sufficient model for fin and limb growth, implicating Msx genes in the embryonic outgrowth of fin rays. Targeted knockdown of *msxB* inhibits fin ray outgrowth, demonstrating that the gene is integral to proliferation as opposed to simply demarcating the distal blastema [54].

Taking the role of *Msx1* into account, it is likely that the observed differences in expression level between the little skate and cownose ray are relevant to phenotypic differences. Swenson et al. [5] found that *Msx1* was not differentially expressed in cownose ray pectoral fins, but there was uniform native expression. For *Msx1* in the little skate, expression is differentially expressed anteriorly. In order for anterior and posterior expression, as quantified by RNA-seq, to be equivalent in the cownose ray, total anterior expression must be diminished. This could be accomplished via weakening expression throughout the domain, restricting the domain of expression, or interrupting the domain of expression. Given that the cephalic fins are separated by a region of the pectoral fin in which fin rays are markedly reduced in length, with a total lack of fin rays at the center of the notch (excluding some members of the genus *Myliobatis*, which retain some diminutive, unbranched fin rays throughout the notch region; [5, 7]) and the role of *Msx1* in fin ray outgrowth, an interruption at the notch region is the most likely cause of the non-differential expression.

### Evolution of cephalic fins in myliobatids via pectoral fin domain splitting may have co-opted an ancient program resulting in clasper/pelvic fin domain splitting in cartilaginous fishes

Swenson et al. [5] found striking similarities between the development of the cephalic limbs and the claspers. Claspers are paired tubular copulatory organs formed from the posterior pelvic fins of male chondrichthyans, used for internal fertilization [55]. The oldest known vertebrate copulatory organs, claspers represent the second of only two known examples of domain splitting in vertebrate limbs resulting in seemingly separate structures that carry out disparate functions [5, 29]. Aside from being morphologically similar in that both structures form when a paired fin is divided into two domains, the claspers are also supported by fin rays and transiently resemble the cephalic fins early in development. Furthermore, the developing claspers and cephalic fins share enrichment of at least five genes unique to these fin regions, including *Hand2*, *Sall1*, *Ntrk2*, *AR*, and *HoxA13* [5, 29]. These observations beg the question: could the cephalic fins in myliobatid rays have evolved via the co-opting and redeployment of genetic pathways associated with clasper development? None of the candidates implicated in domain splitting of the pectoral fins were found to be enriched in the claspers. However, this could be because while the notch specifying the cephalic lobes had formed at the stage in which tissue samples were taken, the divot specifying the claspers had not yet begun to take form [5].

None of the cownose ray embryos available to us were able to be diagnosed male as clasper development had not initiated in any embryos available to us, but ISH was performed on many male little skate embryos. We found that expression of both *Msx1* and *Lhx2* was strongly associated with the claspers as well as with the anterior cephalic fins. In the little skate, these genes are differentially expressed in the anterior pectoral fins, meaning that the total expression in the anterior is greater than the total expression in the posterior of the fin. In the cownose ray, these genes are not differentially expressed, meaning that total expression in both halves of the fin is equal [5]. Interruption of these genes at the notch in cownose ray embryos would reduce total anterior pectoral fin expression, bringing the value to equal posterior pectoral fin expression, resulting in a non-differential relationship.

Although we could not detect *Dkk1* expression in little skates using ISH or examine its expression in the claspers of male cownose rays, we propose that the claspers are formed though a mechanism that parallels that of the cephalic fins—division of the AER via *Dkk1* expression. This is supported by the pattern of *Axin2* expression in little skate pelvic fins. In females, posterior expression is continuous around the posterior lobe of the fin, whereas in males, expression at the notch is interrupted in the same manner as in pectoral fins treated with DKK1 beads, suggesting a division of the AER. These data bring us to the conclusion that cephalic fin development involves a redeployment of the mechanism underlying the development of claspers, linking the only two known examples of domain splitting in vertebrates by a shared mechanistic basis.

## Conclusions: the evolution and development of cephalic fins is tied to an ancient mechanism for shaping distal fin/limb morphologies

We have demonstrated that DKK1 shapes pectoral fins via disruption of the AER and inhibition of fin ray outgrowth. *Dkk1* expression in a representative myliobatid is associated with the evolution and development of cephalic fins. Given these results and the parallel function of *Dkk1* in inhibiting teleost fin ray growth and interrupting the AER in tetrapod limbs, we propose that a DKK1 “brake pedal” and a Wnt-dependent AER “gas pedal” together form an ancient mechanism for shaping distal fin/limb morphologies that dates back to the origin of jawed vertebrates. In addition, cephalic fins evolved via a redeployment of the developmental network underlying the evolution of claspers in cartilaginous fishes, the only other known example of domain splitting in vertebrate limbs.

## Supplementary Information

Supplementary Material 1.

## Data Availability

No datasets were generated or analysed during the current study.

## Abbreviations

AER: Apical ectodermal ridge
APF: Anterior pectoral fin
cDNA: Complimentary deoxyribonucleic acid
CL: Clasper
CR: Crura
ISH: In situ hybridization
PBS: Phosphate-buffered saline
PCR: Polymerase chain reaction
PFA: Paraformaldehyde
PPF: Posterior pectoral fin
RNA: Ribonucleic acid
RT-PCR: Reverse transcription polymerase chain reaction
ZPA: Zone of polarizing activity
