# Supplementary material for: The evolution of cephalic fins in manta rays and their relatives: functional evidence for initiation of domain splitting and modulation of the Wnt signaling pathway in the pectoral fin AER of the little skate
Source: EvoDevo. 2024 Dec 27;15:17. doi: 10.1186/s13227-024-00233-3 (PMC11681717; doi:10.1186/s13227-024-00233-3)
Supplement: Supplementary file 1 — Supplementary Material 1. [file 13227_2024_233_MOESM1_ESM.docx]

**Supplemental Information**


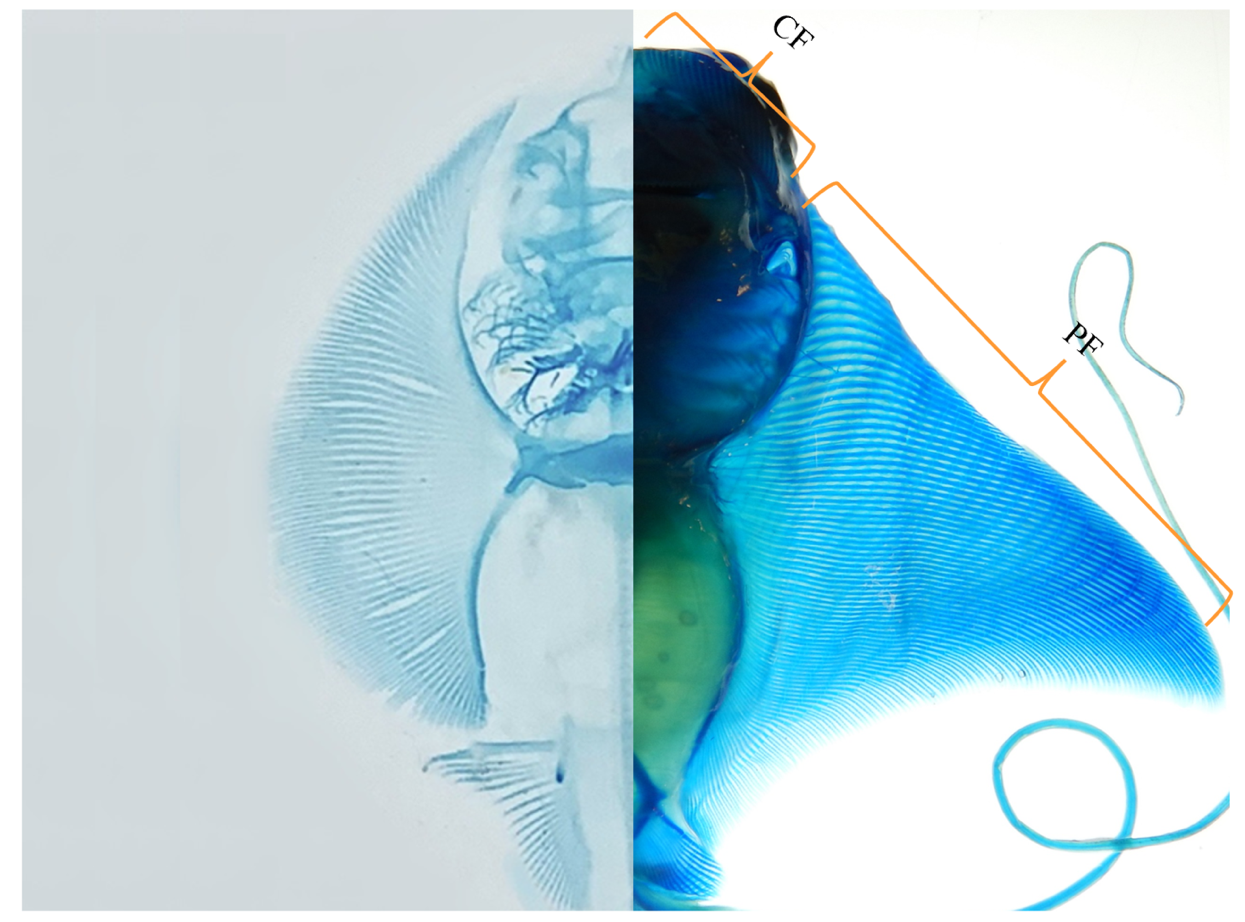


**SI Figure 1. Variation in pectoral fin morphology between the little skate and a myliobatid ray with cephalic fins, which evolved by splitting the pectoral fin into two distinct domains, via interruption of fin ray outgrowth.** The adult skeletal pectoral fin phenotype of the cownose ray (right; *Rhinoptera bonasus*) exhibits a dramatically remodeled body plan; evident when contrasted with the little skate (left; *Leucoraja erinacea*). Brackets indicate the cephalic fin (obscured in this view by layers of dense tissue) and the pectoral fin, separated by the “notch” region defined by reduced fin ray length. Note the A/P asymmetry in the little skate and the derived morphology of the cownose ray with a high aspect ratio and redistribution of fin rays posteriorly, with thicker fin rays in the anterior pectoral fin (following Hall et al. 2018). CF = cephalic fin; PF = pectoral fin.

**
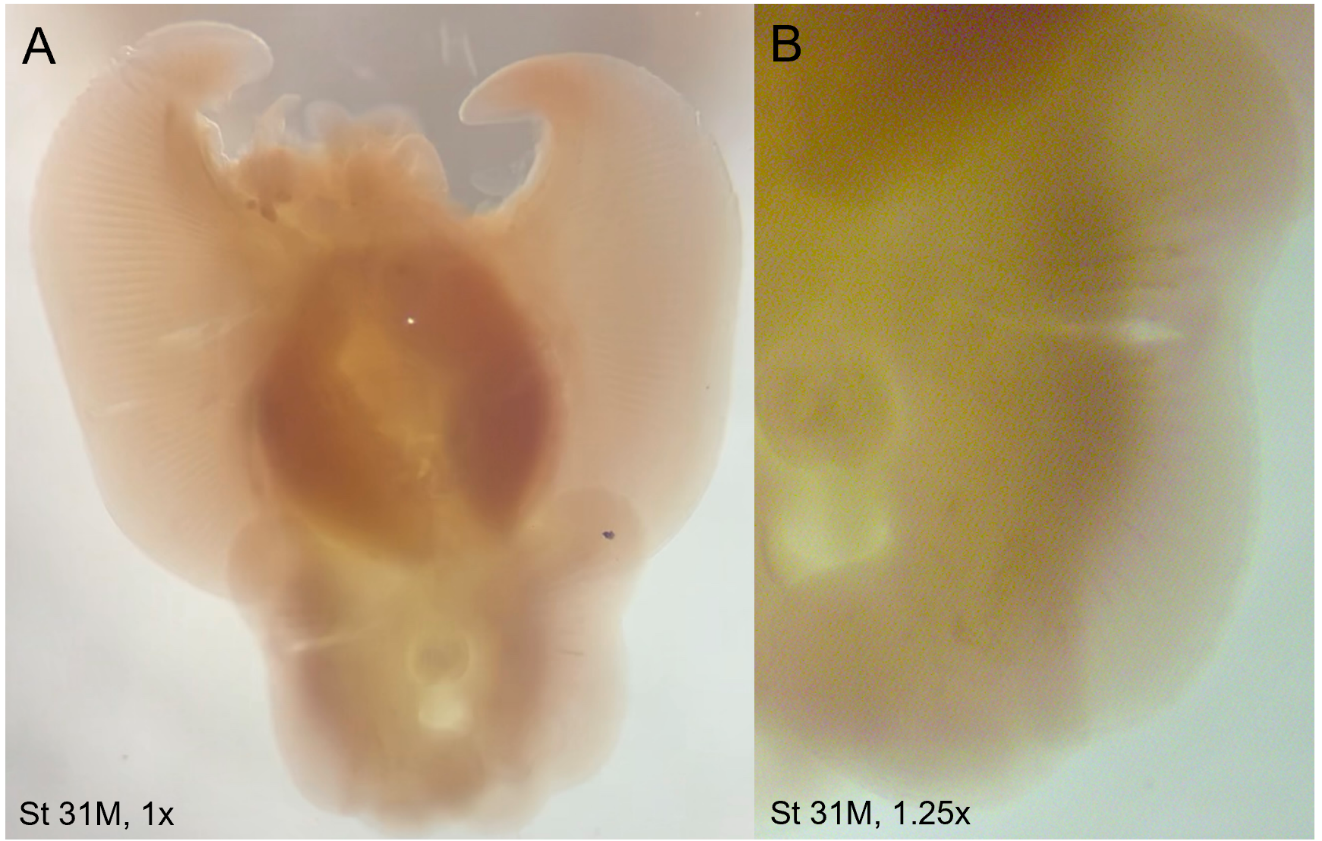
**

**SI Figure 2.** A male little skate embryo at stage 31 that has been treated with the sense mRNA ISH probe for the gene *Msx1*, showing no staining. (**A**) The ventral view of a male little skate embryo at stage 31. Head, tail, and body tissue have been dissected to improve visibility. No expression or staining is detected. (**B**) The ventral view the pelvic fin of a male little skate embryo at stage 31. No expression or staining is detected.


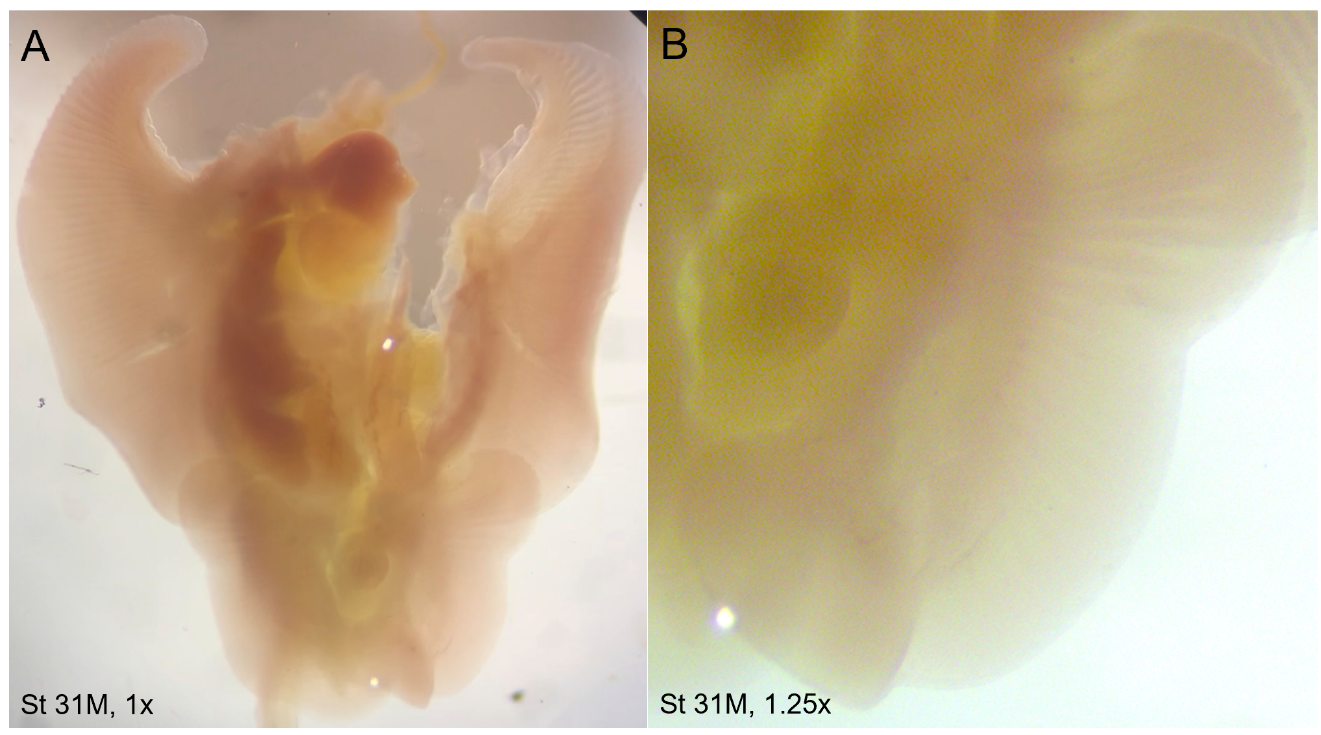


**SI Figure 3.** A male little skate embryo at stage 31 that has been treated with the sense mRNA ISH probe for the gene *Lhx2*, showing no staining. (**A**) The ventral view of a male little skate embryo at stage 31. Head, tail, and body tissue have been dissected to improve visibility. No expression or staining is detected. (**B**) The ventral view the pelvic fin of a male little skate embryo at stage 31. No expression or staining is detected.

**
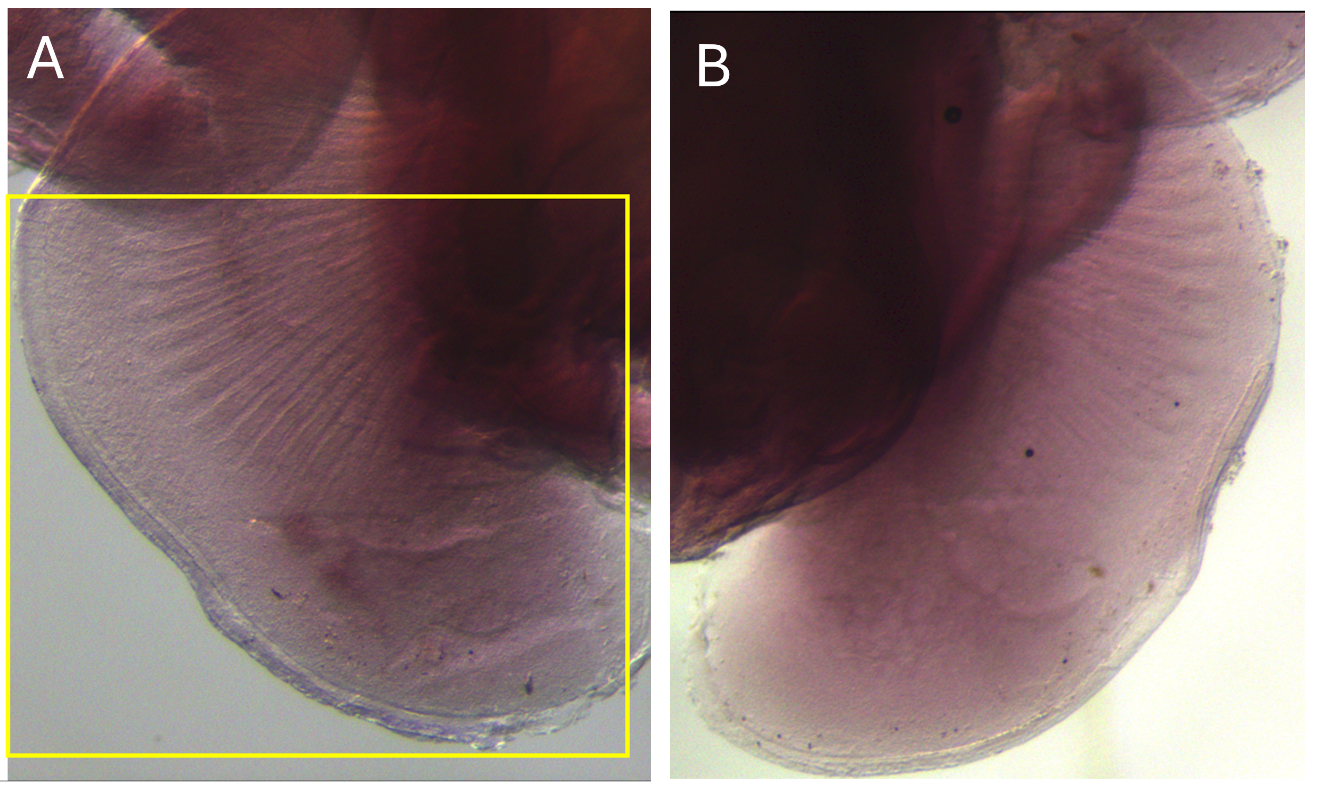
**

**SI Figure 4.** Pelvic fins of cownose ray embryos at stage 3 that have been treated with the antisense (**A**) and sense (**B**) probes for *Dkk1*. Sex at this stage cannot be determined, as the claspers development is not initiated in males. Yellow box denotes region where faint expression can be seen around the perimeter of the pelvic fin.
